# Supplementary material for: MetaRibo-Seq measures translation in microbiomes
Source: Nat Commun. 2020 Jun 29;11:3268. doi: 10.1038/s41467-020-17081-z (PMC7324362; doi:10.1038/s41467-020-17081-z)
Supplement: Supplementary file 10 — Supplementary Data 7 [file 41467_2020_17081_MOESM10_ESM.zip › File2/Confidence_High_Taxonomy/77363_out.krona.html]

Javascript must be enabled to view this page.

members
magnitude
magnitudeUnassigned
count
unassigned
taxon
rank

77363\_out

3

2
3
superkingdom

phylum
1239
3

186801
3
class

3
186802
order

family
3
186803

genus
572511
1

species
1
33039

SRS142712\_contig\_number\_818

1506553
2
genus

species
2
1512

SRS012273\_contig\_number\_contig-100\_19156.50103SRS146888\_contig\_number\_12260
